# Combined Administration of Vitamin D_3_ and Geniposide Is Less Effective than Single Use of Vitamin D_3_ or Geniposide in the Treatment of Ulcerative Colitis

**DOI:** 10.3389/fphar.2021.714065

**Published:** 2021-09-28

**Authors:** Yingyu Lu, Jianqiang Chen, Xueling He, Shuoxi Xu, Yong-er Chen, Jie Gao, Shaozhen Hou

**Affiliations:** ^1^ School of Pharmaceutical Sciences, Guangzhou University of Chinese Medicine, Guangzhou, China; ^2^ The First Affiliated Hospital of Shantou University Medical College, Shantou, China

**Keywords:** ulcerative colitis, vitamin D, p38 MAPK, vitamin D receptor, geniposide (GE)

## Abstract

With the increasing incidence of ulcerative colitis (UC) in China, Chinese medicinal herbs or relatively active compounds are widely applied in treating UC. These medicines may be combined with other therapeutic agents such as vitamin D_3_. Nevertheless, the efficacy of these combinations for UC is unclear. Geniposide is an active component in many Chinese herbal medicines. It could ameliorate dextran sulfate sodium (DSS)–induced colitis in mice. This study was designed to determine the efficacy and mechanism of the single use and combination of geniposide and vitamin D_3_ on a mouse model of acute colitis. Data showed that a single administration of geniposide (2 mg/kg) or vitamin D_3_ (4 IU/day) could significantly improve the symptoms of UC and relieve colon damage. Geniposide and vitamin D could significantly decrease the levels of TNF-α and IL-6 in serum and colon, and increase the level of IL-10 in the colon. However, the combined treatment of geniposide (2 mg/kg) and vitamin D_3_ (4 IU/day) exerted less beneficial effects on UC in mice, indicating by less improvement of UC symptoms, colon damage, and inflammatory infiltration. The combination only downregulated the level of TNF-α in serum and IL-6 in the colon. Our data further demonstrated that geniposide could inhibit the activation of p38 MAPK and then restrict the vitamin D receptor signaling stimulated by vitamin D_3_. These results implied that the combination of geniposide and vitamin D_3_ might not be an ideal combined treatment for acute colitis, and the combination of vitamin D supplementary and geniposide (or herbal medicines rich in geniposide) need more evaluation before being applied to treat UC in clinic.

## Introduction

Inflammatory bowel disease (IBD) is an intestinal disease including Crohn disease (CD) and ulcerative colitis (UC) (Cohen et al., 2019; Piovani et al., 2019). IBD is attracting more concerns than before owing to the high morbidity and relapse rate (Windsor and Kaplan, 2019a). Single therapeutic medicine is not sufficient to treat UC (Benchimol et al., 2019; Bertani et al., 2020; Yamamoto et al., 2020). Combined therapies are often carried for a better outcome. In recent years, the incidence of IBD is on a rapid rise in newly industrialized countries, such as China and other Asian countries (Kaplan and Ng, 2017; Kaplan and Windsor, 2021). Thus, more and more traditional Chinese medicinal herbs are used in the treatment for IBD (Ganji-Arjenaki and Rafieian-Kopaei, 2019; Ananthakrishnan et al., 2020). Some products extracted from Chinese medicinal plants or herbs have been developed as important complementary treatments for UC.

Patients with IBD always have vitamin D deficiency which is considered as a risk factor for the incidence of IBD (Abreu-Delgado et al., 2016; Chetcuti et al., 2018). Vitamin D_3_ (1,25(OH)_2_D_3_) has been widely recommended as an assistant therapy for preventing and alleviating UC (Parian and Limketkai, 2016). Vitamin D activates vitamin D receptor and then regulates a series of gene and protein expression (Froicu and Cantorna, 2007; Bikle, 2014; Christakos et al., 2016). *Gardenia jasminoides* Ellis (*G. jasminoides*) is a traditional Chinese herbal medicine recorded in Chinese pharmacopoeia (2015 edition) with functions of discharging fire and clearing heat, and can treat intestinal diseases such as colitis and hematodiarrhea (Oh and Lim, 2006; Xu et al., 2017). *G. jasminoides* contains large amount of geniposide, which possesses various bioactivities involving anti-inflammation, anti-thrombus, and anti-apoptosis properties (Lee et al., 2013; Zhang et al., 2013; Wang et al., 2015; Jiang et al., 2016; Wang et al., 2018). Some reports revealed that geniposide could alleviate colitis (Xu et al., 2017; Zhang et al., 2017), which might partly work through inhibiting p38 MAPK signaling (Liu et al., 2010; Cheng et al., 2019). Some reports have demonstrated that p38 MAPK signaling pathway could alternate VDR expression (Gill et al., 2002; Sun et al., 2012; Anbazhagan et al., 2018). Thus, we hypothesized that herbal medicines containing rich geniposide might change the beneficial effect of vitamin D_3_ on IBD when they were co-administrated.

Till now, there are few studies concerning about the therapeutic effects of the combination of geniposide or Chinese herbal medicine rich in geniposide and vitamin D_3_ on treating IBD. Therefore, we analyzed the co-effects of geniposide and vitamin D_3_ on a mouse model of UC induced by 4% dextran sulfate sodium (DSS). Our data indicated that combination use of vitamin D_3_ and geniposide was less effective than a single use of vitamin D_3_ or geniposide in the treatment of UC. Geniposide would inhibit p38 MAPK signaling and downregulate the VDR expression stimulated by vitamin D_3_. These results suggested that treating IBD should avoid using the combination of geniposide (or Chinese herbal medicine rich in geniposide) and vitamin D supplement.

## Methods

### Regent and Material

The dextran sulfate sodium (DSS, M.W. 36,000–50,000) was obtained from MP Biomedicals (United States). Geniposide (HPLC >98%, compound CID: 107848) was bought from Yuanye Biotechnology (Shanghai, China). Vitamin D_3_ capsules (1,25-dihydroxyVD_3_) were bought from Haiwang Biological Engineering (Hangzhou, China). The hematoxylin and eosin staining kit was bought from Leagene Biotech (Beijing, China). Biological transparent preparation agent TO was purchased from Kejie Biotechnology (Guangzhou China). Histostain™-Plus Kits and goat anti-mouse IgG/Alexa Fluor 488 were obtained from Biosynthesis Biotechnology (Beijing, China). Neutral gum was bought from Labgic Technology (Beijing, China). DAB substrate kit, anti-rabbit IgG (H + L), F (ab')2 Fragment (Alexa Fluor^®^ 555 Conjugate), and p-p38 MAPK (Thr180/Tyr182) were obtained from Cell Signaling Technology (United States). RIPA buffer, phenylmethanesulfonyl fluoride (PMSF) inhibitors, phosphates inhibitors, and SDS-PAGE loading buffer were provided by CoWin Biosciences (Jiangsu, China). Bovine serum albumin (BSA) fraction V was bought from Saiguo Biotech (Guangzhou, China). PVDF membrane (size: 0.45 μm) and Immobilon™ Western Chemiluminescent HRP Substrate were purchased from Millipore Corporation (Billerica, United States). One-step gel preparation kit (8%) was obtained from Fude Biological technology (Hangzhou, China). Na^+^/H^+^ exchange (NHE3) rabbit polyclonal antibody and GAPDH mouse monoclonal antibody were provided by Proteintech Group, Inc. (Rosemont, United States). Goat anti-mouse IgG (H + L) HRP, p38 MAPK antibody, vitamin D receptor (VDR) antibody, and goat anti-rabbit IgG (H + L) HRP were purchased from Affinity Biosciences (United States). DAPI was bought from Sigma-Aldrich (Louis, United States). Elisa kits (mouse TNF-α, IL-6, and IL-10) were bought from MEIMIAN BIOLOGY (China)

### Animals

66 Kunming male mice, weighing 14–16g, were obtained from Guangdong Experimental Animal Center (License Number: SCXK 2013-0002) and housed in Laboratory Animal Services Centre at Guangzhou University of Chinese Medicine (License Number: SYXK 2018-0085). All mice were fed under 25°C temperature and 55 ± 10% humidity with 12 h light/dark cycles. They had access to food and water freely. The study was guided under institutional and National Institutes of Health (NIH) guidelines for humane animal use. The experimental protocols were permitted by the Animal Ethics Committee of Guangzhou University of Chinese Medicine (License Number: 20200605004).

### Establishment of UC Model and Treatment

**FIGURE 1:**
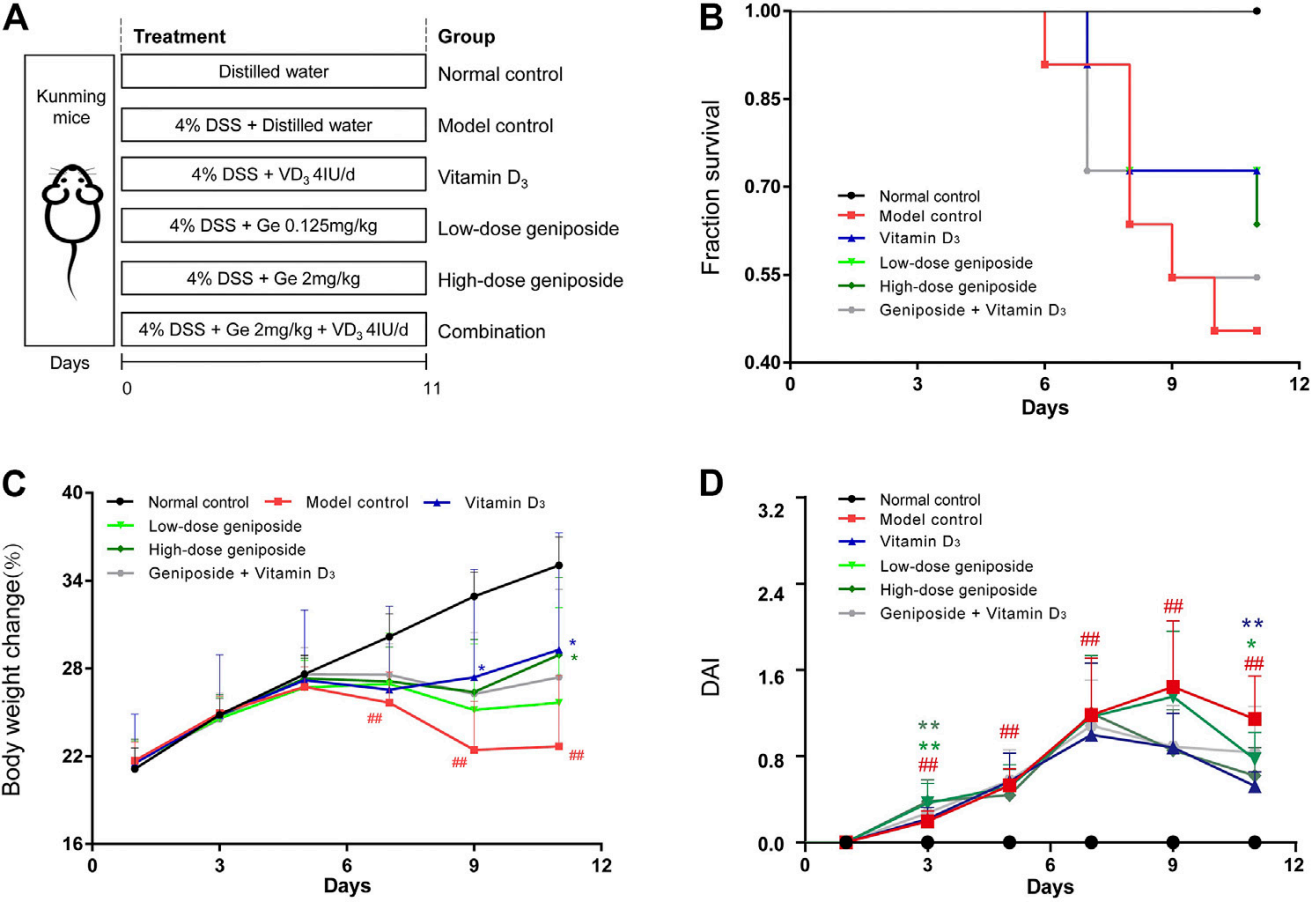
Combination of vitamin D_3_ and geniposide exerted less beneficial effects on improving the symptoms and the survival of UC mice. **(A)** Experimental design protocol. **(B)** Survival curve. **(C)** Body weight change. **(D)** Disease activity index. ^*#*^ *p* < 0.05, ^*##*^ *p* < 0.01, vs. the normal control group; *p < 0.05, **p < 0.01, vs. the model control group. n = 11 per each group.

As showing in Figure 1A, after 7-days adaptive feeding, all mice were randomly grouped into normal control group (NC), model control group (DSS), vitamin D_3_-treated group (VD_3_), low-dose geniposide-treated group (Ge L), high-dose geniposide-treated group (Ge H), and co-administration group (COMB). Drug treatments once daily were started along with colitis establishment. 4% DSS was used as daily drinking for inducing UC in mice except for the NC group. Normal control group and model control group were treated with distilled water (i.g.). VD_3_ group was treated daily with 4 IU vitamin D_3_. The low-dose geniposide group was administered with 0.125 mg/kg geniposide (i.g.) and the high-dose group with 2 mg/kg (i.g.). The combination group was treated with 4 IU/d vitamin D_3_ and 2 mg/kg geniposide (i.g.). Daily body weight, diarrhea, hematochezia, and animal statues in each group were recorded. At the end of experimental modeling, the colonic length and thickness were measured. Then, colons were partially stored at −80°C and immersed in 4% buffered neutral formalin for following testing and examination.

### Disease Activity Index (DAI)

Mice showed diarrhea, hematochezia, and loss of weight after 11 days of drinking 4% DSS, indicating the establishment of UC. Two researchers evaluated DAI and were experimental blinded. During the experimental modeling, referring to Tohru’s (Funakoshi et al., 2012) method, DAI values were daily recorded and calculated according to body weight, severity of diarrhea, and hematochezia with some adjustment. Percentage of weight loss (A): 0 meant no weight loss; 1 meant a 0–5% loss; 2 meant a 5–10% loss; 3 meant a 10–15% loss; and 4 meant over 15% loss. States of fecal (B): 0 meant normal feces; 1–2 meant wet and soft feces; and 3–4 meant loose feces. Severity of hematochezia (C): 0–4, 0 was normal or occult blood; 1–2 was dim blood; and 3–4 was obvious blood. The DAI score was calculated according to (A + B + C)/3.

### Histopathology of Colon Tissues

After being immersed in 4% paraformaldehyde, colon tissues were dehydrated, embedded in paraffin, cut into slices, and then dewaxed and stained with hematoxylin and eosin. Histological symptoms of UC are inflammatory infiltration, vascular changes, and epithelial damage. Histopathological scores (Lu et al., 2018): 0 meant normal tissues; 1–2 meant epithelial less damage and mild mucous membrane inflammation characterized by mononuclear cells; 3–4 meant moderate inflammation characterized by monocyte, a small quantity of neutrophils with crypt glands being far from basement membrane, and decreased mucin in goblet cells; 5–6 meant severe inflammation characterized by submucosal mononuclear and neutrophils infiltrations, crypt abscesses, decreased mucin, epithelial damage and ulceration; and 7–8 meant crypt disappearance and serious mucosal inflammation characterized by neutrophils.

### Western Blot Analysis of p38 MAPK, p-p38 MAPK, and VDR Expression

Colon tissue samples were homogenized at 4°C and 60 Hz for 2 min. Then, the protein was separated and mixed with loading buffer by the ratio of 4:1. Protein samples were separated by 8% SDS-PAGE gel and transferred onto a PVDF membrane. After being blocked, the membranes were incubated with different primary antibodies (p38 MAPK, p-p38 MAPK, VDR, and GAPDH) diluted in 3% BSA at 4°C overnight. Then, after being incubated with secondary antibodies at room temperature, protein bands were shown by ECL kit. Finally, the bands were quantitatively analyzed by ImageJ software.

### Immunohistochemical Staining of p-p38 MAPK in the Colon Tissues

According to the protocol of the IHC Kit, dewaxed sections of colon sections were soaked in phosphate buffered saline (PBS) for 5 min 2% EDTA-antigen retrieval buffers was used for antigen retrieval. Then, endogenous peroxidase activity was eliminated by 20 min-incubation in 3% H_2_O_2_. After that, sections were blocked with goat serum for 20 min and incubated with p-p38 MAPK antibody diluted in PBS (1:500) at 4°C overnight. These sections were incubated with IgG/Bio for 20 min and further incubated with S-A/HRP for 20 min at room temperature. DAB was added onto each section for 30 s. After being re-dyed, dehydrated, transparentized, and sealed, these sections were observed by microscope and quantitatively analyzed by Image-Pro Plus 6.0 software.

### Immunofluorescence Staining of VDR

According to the method of immunofluorescence staining, VDR antibodies were used as primary antibodies, respectively. Anti-rabbit IgG (H + L), F (ab')2 Fragment (Alexa Fluor^®^ 555 Conjugate) were used as secondary antibodies. Observations with a confocal laser microscope were carried out after 5 min DAPI incubation. The intensity was quantitatively analyzed by Image-Pro Plus 6.0 software.

### Detection of Inflammatory Cytokines in Serum and Colon Tissue

Colon tissue samples were put in PBS and homogenized at 4°C and 60 Hz for 2 min. Then samples were centrifuged in a cryogenic centrifuge at 10,000 rpm for 15 min. Blood samples were obtained from each animal at the end of the experimental period. Serum was extracted from blood samples after being centrifuged in a cryogenic centrifuge at 10000 rpm for 15 min. According to the instructions in the ELISA kit, the concentrations of TNF-α, IL-6, and IL-10 in serum and colonic samples were detected.

### Statistical Analyses

Data were expressed as mean ± S.E.M. and were analyzed by the analysis of variance with an ANOVA test. The difference *p < 0.05* was statistical significance.

## Result

### Combination of Vitamin D_3_ and Geniposide Exerted Less Beneficial Effects on Improving the Symptoms of Ulcerative Colitis (UC) Mice

At the end of the experimental period, the mice fed with 4% DSS exerted significant weight loss, severe diarrhea, and hematochezia and mice in the DSS group had lower survival (Figures 1A–D). Both geniposide (0.125 mg/kg and 2 mg/kg) and vitamin D_3_ (4 IU/day) could improve the survival of UC mice, while the COMB group treated with geniposide (2 mg/kg) and vitamin D_3_ (4 IU/day) had less improvement on the survival than VD_3_ group, GE H group, and GE L group (Figure 1B). Vitamin D_3_ could remarkably alleviate the severity of body weight loss and loose and bloody feces caused by UC in VD_3_ group (Figures 1C,D). High-dosage geniposide could improve the weight of mice in GE H group (Figures 1C,D). The combination of geniposide and vitamin D_3_ did not exert more beneficial effects on these symptoms in COMB group (Figures 1C,D). It was indicated that a combination of vitamin D_3_ and geniposide did not have more beneficial effects than single administration of vitamin D_3_ and geniposide. The difference between improvement in colon damage of combination and single treatment needs more investigation.

### Combination of Vitamin D_3_ and Geniposide Did Not Improve the Length of the Colon in UC Mice

**FIGURE 2:**
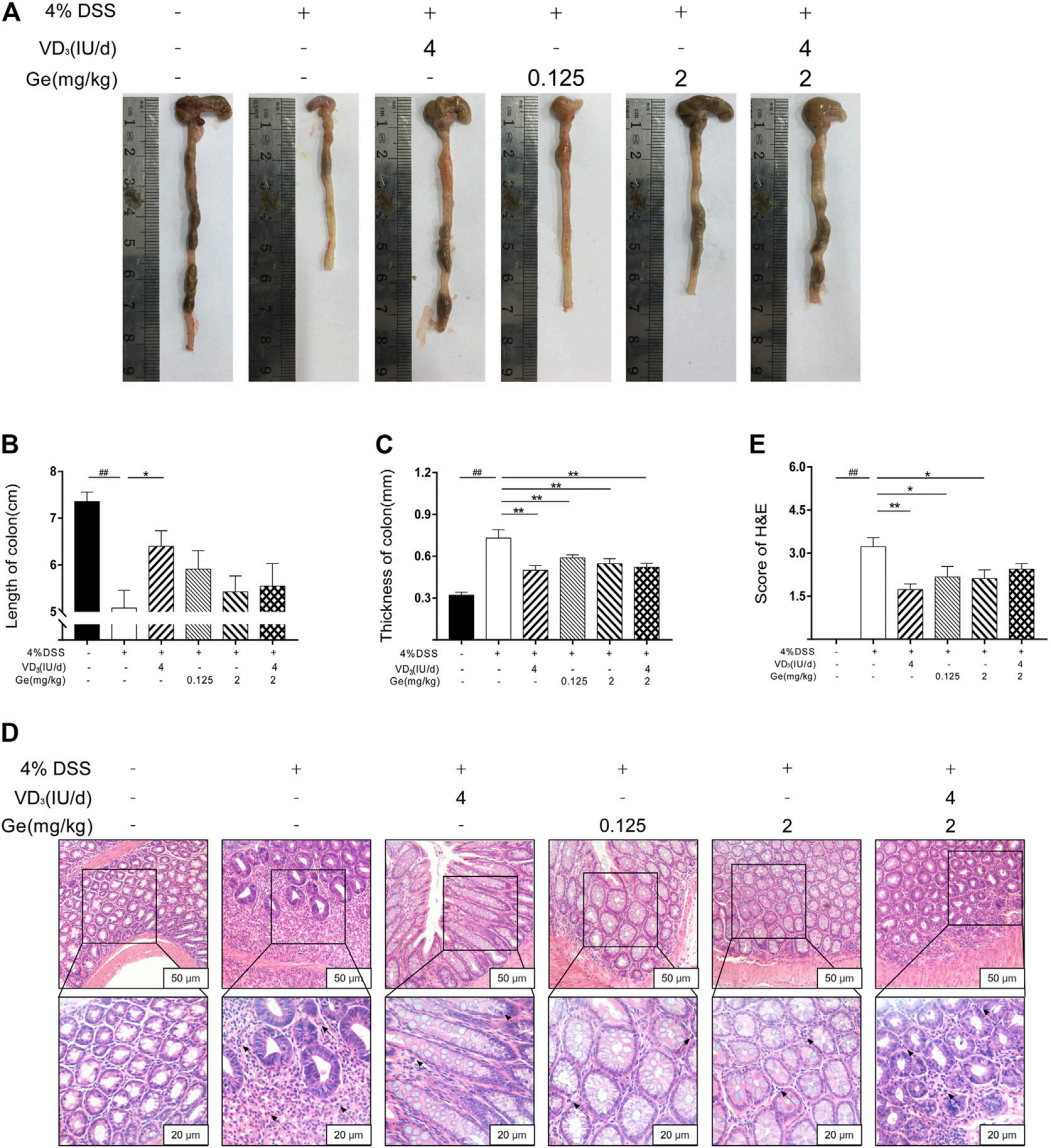
Combination of vitamin D_3_ and geniposide could not improve the length of colon in UC mice. **(A)** Representative colon of each group. **(B)** Length of colon. **(C)** Thickness of colon. **(D)** Representative colonic tissue of each group (200× and 400×). **(E)** H&E score. ^*##*^ *p* < 0.01, vs. the normal control group; *p < 0.05, **p < 0.01, vs. the model control group. n = 11 per each group.

Colon would be significantly shortened and the thickness was significantly increased by DSS-induced colitis (Figures 2A–C). 4 IU/day of vitamin D_3_ could significantly improve the length and thickness of the colon in the VD_3_ group (Figures 2A–C). Geniposide (0.125 mg/kg and 2 mg/kg) as well as a combination of vitamin D_3_ (4 IU/day) and geniposide (2 mg/kg) could only alleviate the thickness but could not improve the length of the colon (Figures 2A–C). It was implied that geniposide might restrict the effect of vitamin D_3_ on improving colonic injury in UC mice. The colons of mice with UC exhibited significant inflammatory infiltration and disorder of cell arrangement (Figure 2D; black arrows). These damages could be remarkably alleviated by vitamin D_3_ or geniposide, respectively, but the combination of vitamin D_3_ and geniposide did not have any significant improvement in inflammatory infiltration (Figures 2D,E; black arrows), which meant the combination of vitamin D_3_ and geniposide might have less anti-inflammatory effect.

### Combined Treatment of Vitamin D_3_ and Geniposide Exerted Poor Improvement on the Levels of Inflammatory Cytokines

**FIGURE 3:**
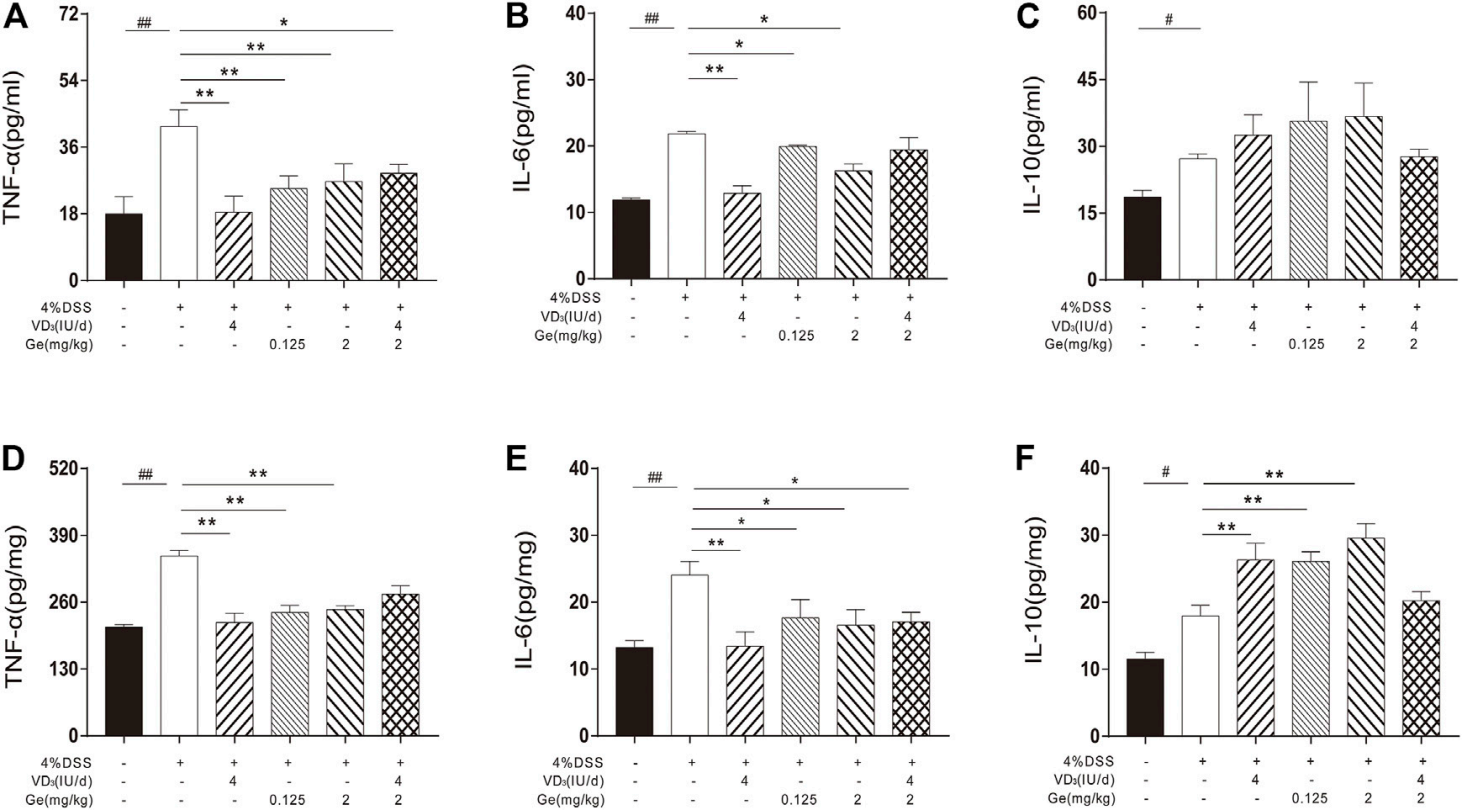
Combined treatment of vitamin D_3_ and geniposide exerted poor improvement on the levels of inflammatory cytokines. **(A)** Serum level of TNF-α. **(B)** Serum level of IL-6. **(C)** Serum level of IL-10. **(D)** Tissue level of TNF-α. **(E)** Tissue level of IL-6. **(F)** Tissue level of IL-10. ^*#*^ *p* < 0.05, ^*##*^ *p* < 0.01, vs. the normal control group; *p < 0.05, **p < 0.01, vs. the model control group. n = 8 per each group.

In order to investigate the anti-inflammatory effect of a combination of vitamin D_3_ and geniposide, the inflammatory cytokines in serum and colon tissue were detected. In both serum and colon tissue, the levels of TNF-α, IL-6, and IL-10 were significantly increased in UC mice (Figures 3A–G). Either vitamin D_3_ (4 IU/day) or geniposide (0.125 and 2 mg/kg) could significantly downregulate the levels of TNF-α and IL-6 in the serum and colon tissue, and significantly upregulate the level of IL-10 in colon tissue (Figures 3B–G). Combination of vitamin D_3_ (4 IU/day) and geniposide (2 mg/kg) could only decrease the levels of TNF-α in serum and IL-6 in colon tissue (Figures 3B–G). It was suggested that the combination of vitamin D_3_ and geniposide did not show better anti-inflammatory effect than single administration of vitamin D_3_ or geniposide.

### Combined Treatment of Vitamin D_3_ and Geniposide Induced Low Level of p-p38 MAPK

**FIGURE 4:**
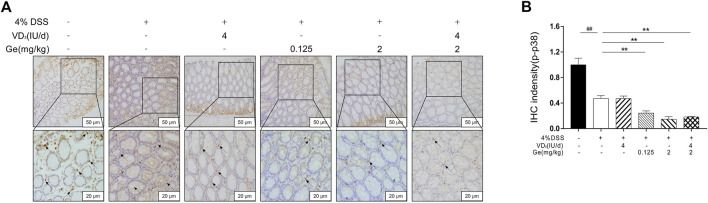
Geniposide inhibited the expression of activated p38 MAPK. **(A)** Representative p-p38 MAPK expression in each group (200× and 400×); **(B)** IHC intensity of p-p38 MAPK. ^*##*^ *p* < 0.01, vs. the normal control group; **p < 0.01, vs. the model control group. n = 6 per each group.

In the present study, the activation of p38 MAPK was significantly decreased in the UC mice when compared to the normal control group (Figures 4A,B). The activation of p38 MAPK was suppressed further in all mice treated with geniposide, including mice in the COMB group (Figures 4A,B). This result was consistent with other reports that geniposide could inhibit the activation of p38 MAPK (Liu et al., 2010; Cheng et al., 2019). These results suggested that geniposide ameliorated DSS-induced colitis through inhibiting the activation of p38 MAPK. Considering the interaction between p38 MAPK and VDR, the expression of VDR needs more investigation in COMB group.

### Combined Treatment of Vitamin D_3_ and Geniposide Induced Low Expression of VDR

**FIGURE 5:**
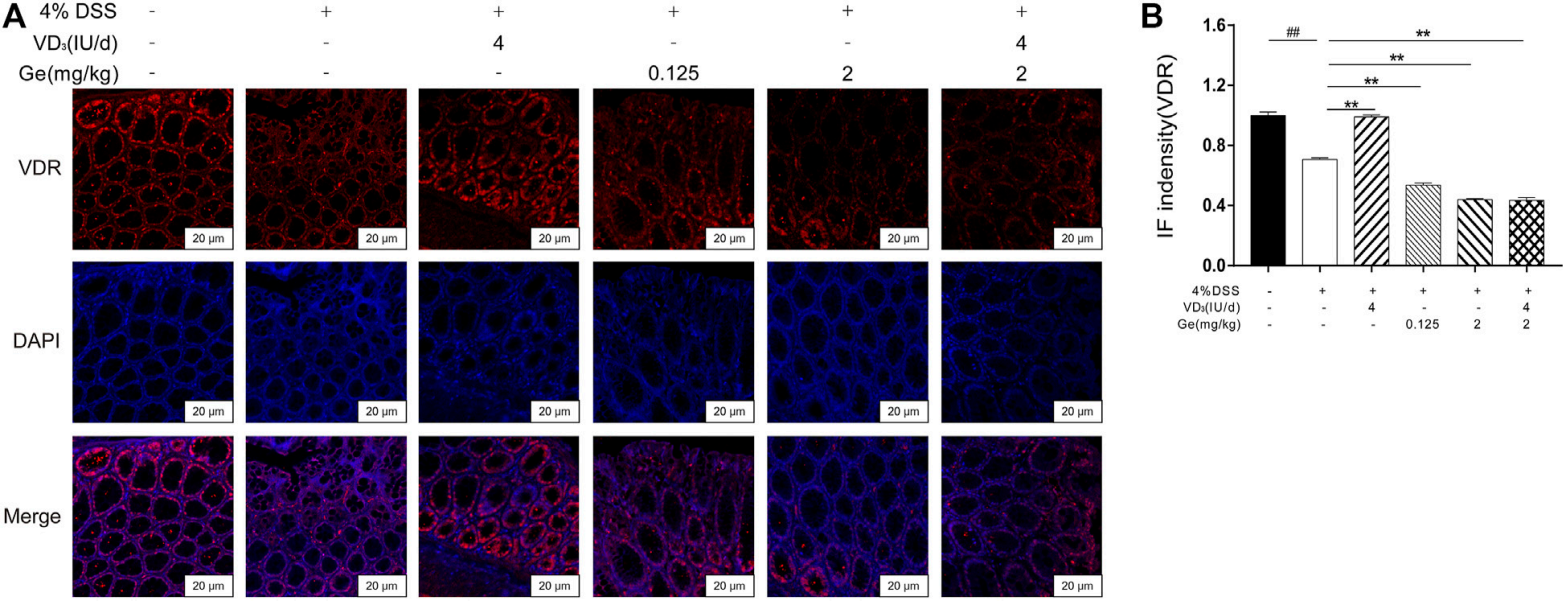
Geniposide inhibited the expression of VDR and attenuated the simulative effect of vitamin D_3_ on VDR. **(A)** Representative VDR expression in each group (400×); **(B)** IF intensity of VDR. ^*##*^ *p* < 0.01, vs. the normal control group; **p < 0.01, vs. the model control group. n = 6 per each group.

Vitamin D_3_ exerts its effects by binding to a specific transcriptional regulation molecule, the VDR (Makishima, 2018; Muralidhar et al., 2019). The expression of VDR significantly decreased in the colon tissue of mice with UC (Figures 5A,B). Vitamin D_3_ could improve the expression of VDR in the colon of mice with UC (Figures 5A,B). The expression of VDR was remarkably inhibited in several geniposide-treated group including the COMB group (Figures 5A,B). These results indicated that geniposide could inhibit the expression of VDR which might suppress the activation of VDR stimulated by vitamin D_3_.

### Combined Treatment of Vitamin D_3_ and Geniposide Induced Downregulation of Activation of p38 MAPK and Expression of VDR by Western Blot

**FIGURE 6:**
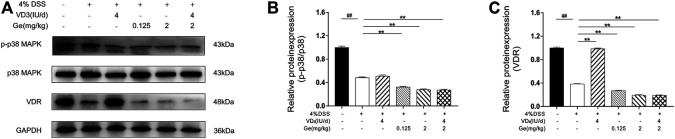
Geniposide attenuated the effects of vitamin D_3_ by inhibiting p38 MAPK pathway and then downregulating the expression of VDR. **(A)** Expressions of p-p38 MAPK, p38 MAPK, and VDR analyzed by Western Blot. **(B)** Relative expressions of p-p38 MAPK and p38 MAPK. **(C)** Relative expression of VDR. n = 3 per each group.

Our data showed that expressions of VDR and activation of p38 MAPK were significantly decreased in colons of UC mice (Figures 6A–C). Geniposide further downregulated the activation of p38 MAPK, indicating the inhibition effect on p38 MAPK pathway of geniposide. It had been demonstrated that decrease of p38 MAPK signaling would reduce the expression of VDR (Sun et al., 2012). Vitamin D_3_ treatment could elevate the expression of VDR in the VD_3_ group. However, VDR expression was significantly reduced in the presence of geniposide (Figures 6A–C). Thus, our data suggested geniposide could attenuate the effect of vitamin D_3_ on stimulating VDR through inhibiting p-p38 MAPK.

## Discussion

Epidemiological data have demonstrated that vitamin D deficiency is correlative with higher risk of IBD (Froicu and Cantorna, 2007; Abreu-Delgado et al., 2016; Limketkai et al., 2017; Chetcuti et al., 2018), thus vitamin D supplements are widely recommended to prevent and cure colitis. In the present study, animal with colitis induced by DSS showed downregulated VDR expression. Current therapies could not always cure IBD thoroughly (Benchimol et al., 2019; Bertani et al., 2020; Yamamoto et al., 2020). The incidence of IBD is rapidly increasing in China (Ananthakrishnan et al., 2020). Chinese medicines might be an optional therapeutic method for patients (Zhang et al., 2013; Kou et al., 2020; Ye et al., 2020). More and more Chinese herbal medicines and relative products might be taken along with vitamin D supplements when treating IBD.

Vitamin D_3_ exerts its bioactivities mainly by activating VDR (Froicu and Cantorna, 2007; Bikle, 2014; Christakos et al., 2016). Many clinical trials reported the beneficial effects of vitamin D supplementation on improving IBD (Arihiro et al., 2019; Emami et al., 2020). In this study, data showed that UC mice could be improved by daily treatment of vitamin D_3_. Vitamin D_3_ upregulated the expression of VDR in colons of UC mice. Vitamin D_3_ could alleviate the symptoms and colon damage and improve levels of inflammatory cytokines in serum and colon in UC mice. These results proved that single administration of vitamin D_3_ had improvement on DSS-induced colitis. Geniposide could ameliorate hematochezia, diarrhea, and weight loss in UC mice, which was consistent with other reports (Liu et al., 2010; Cheng et al., 2019). The activation of p38 MAPK was inhibited and the inflammation of UC mice was alleviated by single treatment of geniposide. Inflammatory cytokines in serum and colon of GE H group and GE L group were improved. These results verified that geniposide could improve colitis by inhibiting p-p38 MAPK signaling. However, the combination of vitamin D_3_ and geniposide exhibited less improvement on survival and shortened colons of UC mice than single treatment of vitamin D_3_ or geniposide. Levels of inflammatory cytokines increased by DSS-induced colitis did not show a greater downregulation in the COMB group when compared to the VD group, GE L group, and GE H group. Only TNF-α in the serum and IL-6 in the colon exerted improvement in the COMB group. Then, it was found that the level of activated p38 MAPK of COMB group was similar to the GE H group. Daily treatment of 4IU vitamin D_3_ did not have significant influence on p38 MAPK activation. It was indicated that vitamin D_3_ might not have impacts on p-p38 MAPK inhibition of geniposide. Considering the different inflammatory cytokines levels of single treatment and combined treatment, vitamin D_3_ might influence the anti-inflammatory effect of geniposide through other pathways. Unexpectedly, VDR expression could not be improved by vitamin D_3_ in the COMB group, and it had a significant downregulation compared to the DSS group. It had been demonstrated that decrease of p38 MAPK signaling would reduce the expression of VDR (Sun et al., 2012). These signs indicated that geniposide-inhibited p-p38 MAPK might inhibit the expression of VDR and then fade the efficacy of vitamin D_3_. In this study, we have demonstrated that geniposide, as an anti-inflammatory compound partially inhibiting p-p38 MAPK, might alter the expression of VDR and weaken the effect of vitamin D supplements on alleviating colitis. This study had confirmed that VDR signaling was restricted in the COMB group, as geniposide would reduce p-p38 MAPK and alternated the expression of VDR stimulated by vitamin D_3_. The co-administration of geniposide or geniposide-rich Chinese medicinal herbs and vitamin D supplement needs more value and cautions for patients with UC.

## Conclusion

The present study proved that a single treatment of vitamin D_3_ or geniposide could ameliorate DSS-induced UC in mice. However, it did not show a better outcome in mice treated with a combination of vitamin D_3_ and geniposide. The potential mechanism of this fading efficacy might be that geniposide could downregulate vitamin D-VDR signaling and reduce the protective effects of vitamin D_3_ through inhibiting p-p38 MAPK.

## Data Availability

The raw data supporting the conclusions of this article will be made available by the authors, without undue reservation.
