# Supplementary figures and images for: Combined Administration of Vitamin D3 and Geniposide Is Less Effective than Single Use of Vitamin D3 or Geniposide in the Treatment of Ulcerative Colitis
Source: Front Pharmacol. 2021 Sep 28;12:714065. doi: 10.3389/fphar.2021.714065 (PMC8505666; doi:10.3389/fphar.2021.714065)

## p38 MAPK

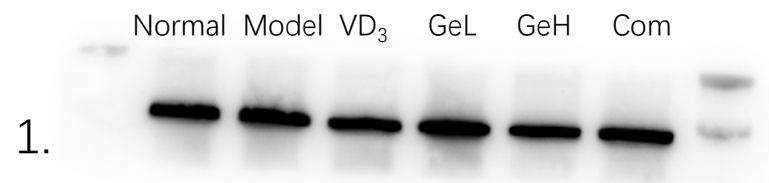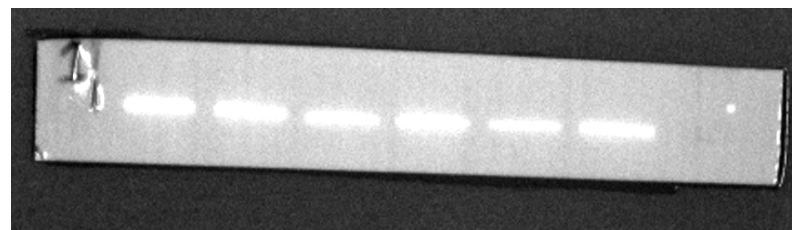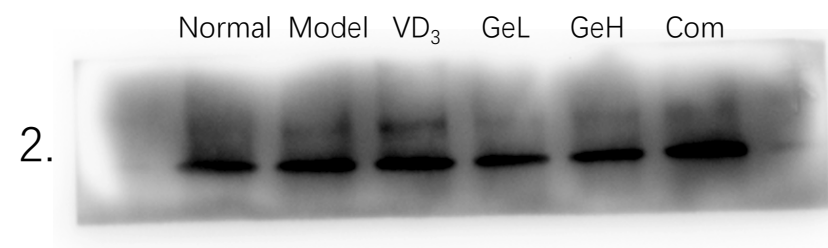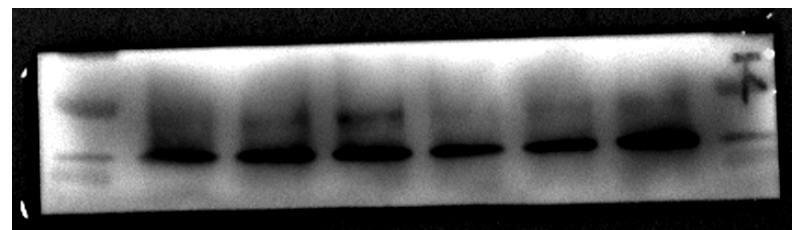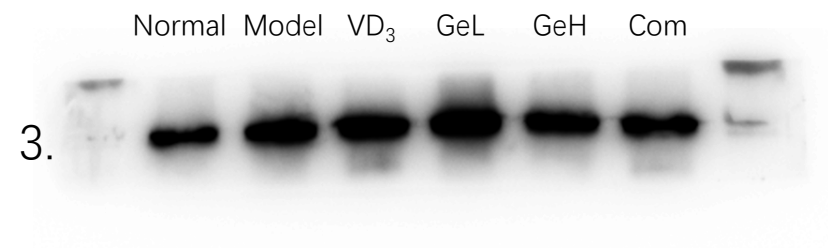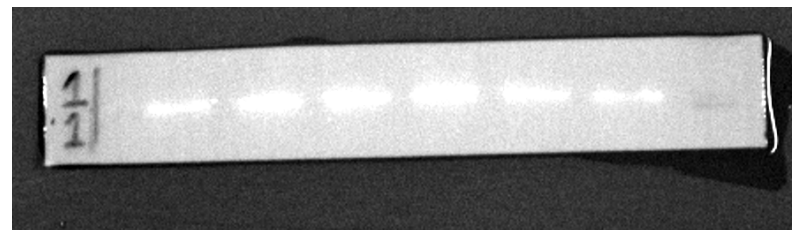

## p-p38 MAPK

1.

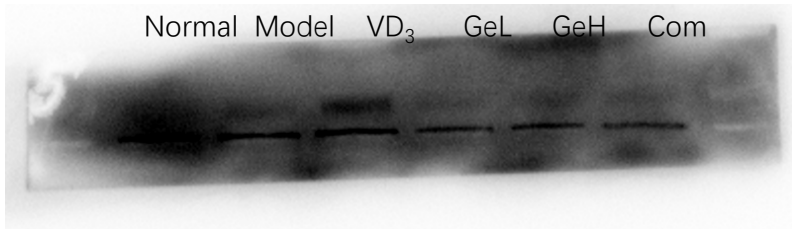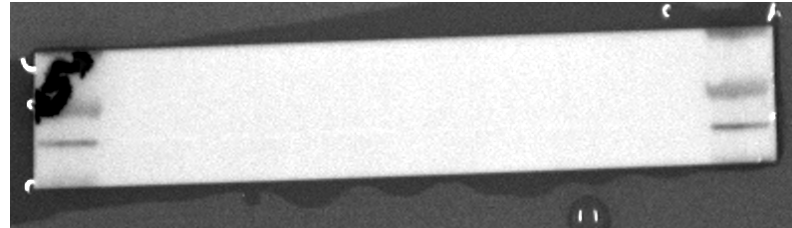

2.

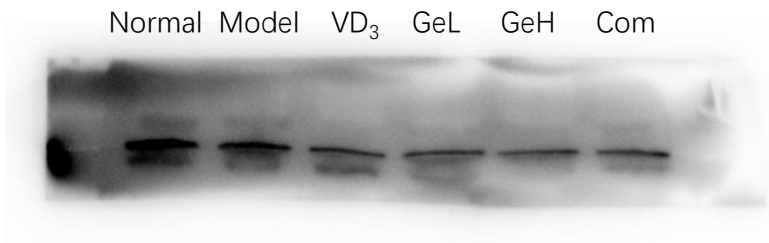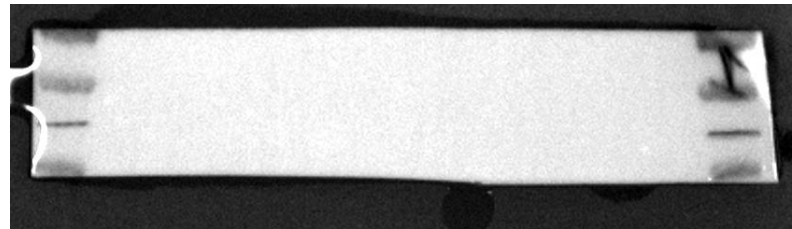

3.

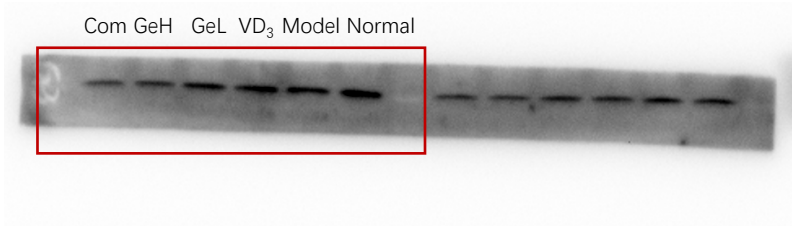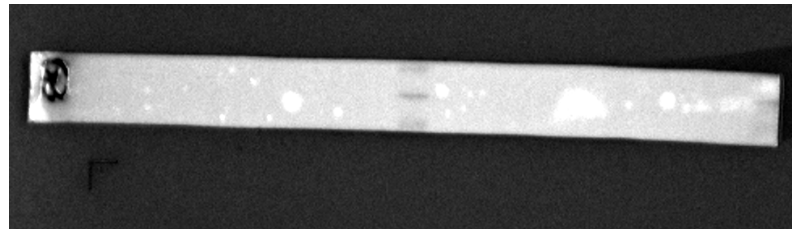

# VDR

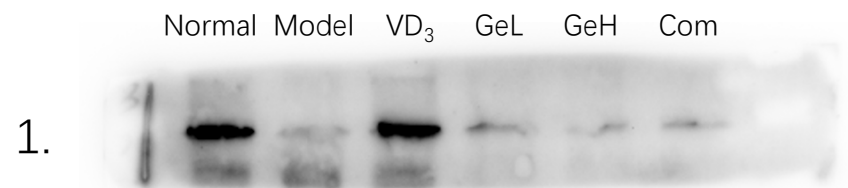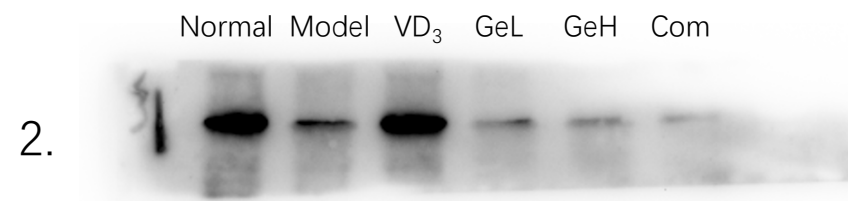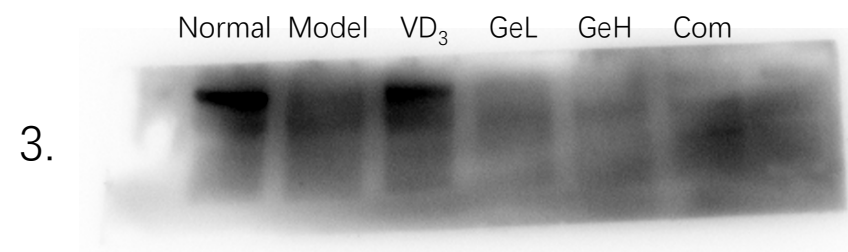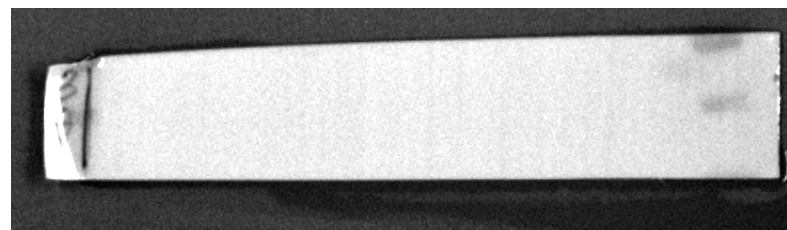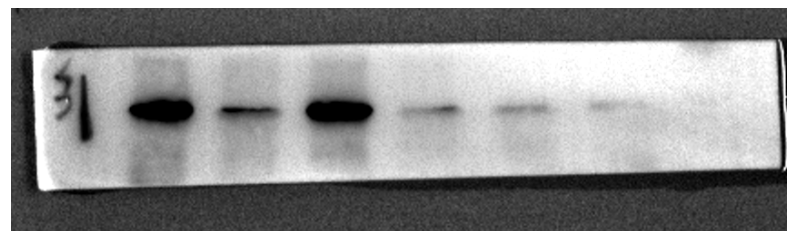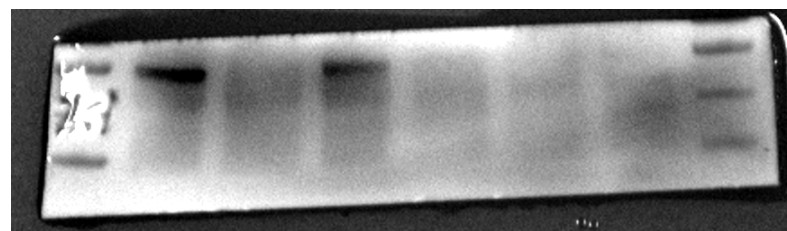

# GAPDH

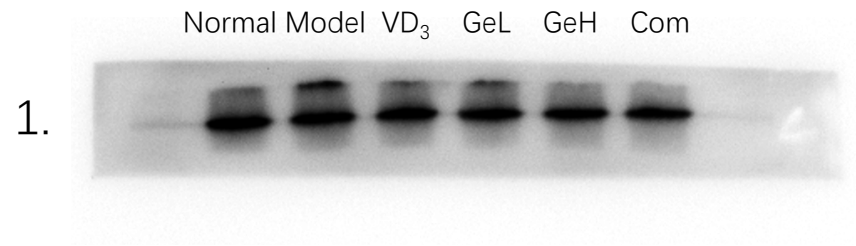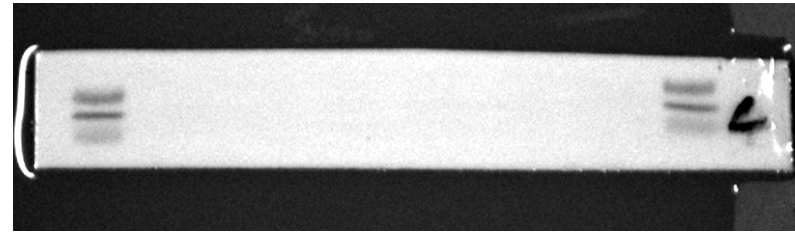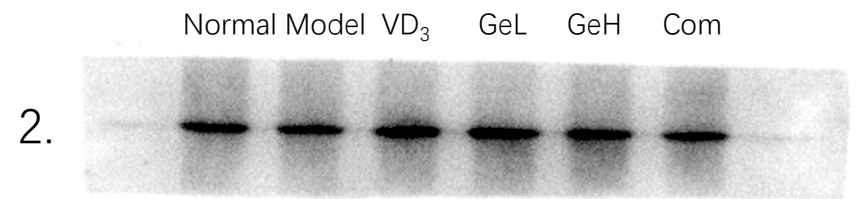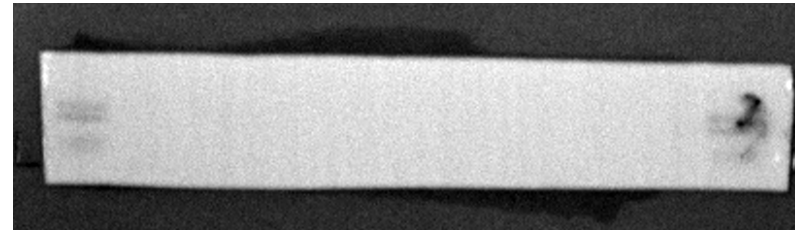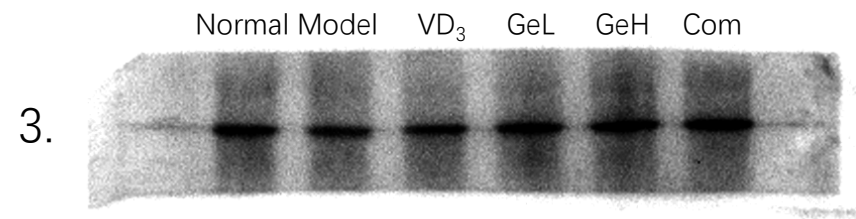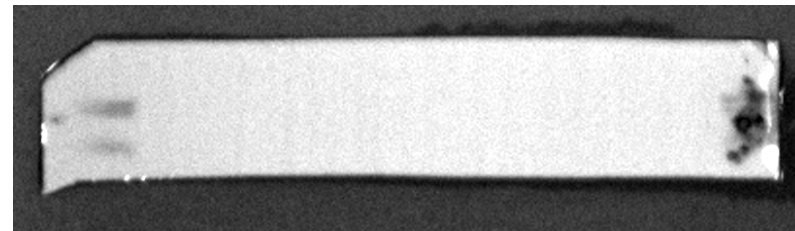

Supplement: Supplementary file 1 [file DataSheet1.PDF]
